# Construction Schedule Management System for Large-Scale Construction Projects Based on Multisensor Network

**DOI:** 10.1155/2022/3003552

**Published:** 2022-09-14

**Authors:** Fanmin Meng, Shaozhi Yu, Jianying Xue

**Affiliations:** ^1^School of Science, North University of China, Taiyuan, Shanxi 030051, China; ^2^Huayuan International Land-Port Group Co., Ltd., Taiyuan, Shanxi 030021, China

## Abstract

As an important task of construction project management, construction schedule management is related to the realization of project period, cost, quality, and other objectives. However, in actual construction, problems such as difficulties in plan implementation and management are often encountered, and schedule delays often occur. Moreover, with the increase in the scale and complexity of modern buildings, the management of construction schedules has brought about larger issues and higher requirements have been put forward for the management level of the construction schedule. The traditional timetable management and scheduling is not highly relevant to the project entity. There is no entity change, the project information loss is serious, the tracking and control of the construction timetable is not in place, there is fine management, and the construction timetable management cannot meet the needs of modern construction. In order to ensure the smooth implementation of projects and objectives, it is urgent to explore new management ideas and methods to promote the smooth progress of construction process management. BIM technology has the advantages of visibility, simulation, adjustment, optimization, and advanced data integration, which helps to make up for the shortcomings of previous schedule management and improve the level of construction schedule management. Based on the research situation of large-scale construction project monitoring system and the analysis of information fusion technology, this paper proposes a large-scale construction project monitoring system scheme based on multisensor network information fusion. Through the investigation of the monitoring content, the composition of the multisensor network is determined. This paper is applicable to large-scale construction projects. Through the research and design of hardware modules, software modules, and fusion algorithms, the data acquisition, transmission, calculation, and display functions of the monitoring system based on multisensor network information fusion are realized, which can effectively monitor the entire process of large-scale construction projects.

## 1. Introduction

With the continuous development of science and information technology, one of the most important means of obtaining external information-sensor technology has also developed rapidly. The acquisition mode of sensor information has gradually moved from the past singles to the direction of mergers and networks. Multisensor network coordinated detection technology is an important field of multisensor network that has received high attention in military, medical, rescue, and other fields. In the current complex and changeable environment, the information provided by a single sensor no longer meets the demand, and it is difficult to complete the task of information collection [1]. Because it is specifically reflected in a limited number of sensors, multiple sensors cannot fully cover the entire target area environment and cannot obtain complete information [2]. In a complex dynamic target environment, the sensor's detection of the target environment has great uncertainty [3]. The lack of communication and collaboration between multiple sensors will result in a high degree of redundancy in acquiring sensor information [4]. Therefore, in order to obtain more accurate environmental information of the target area, multiple sensors are used in real-time target detection to obtain the environmental information of the target area as comprehensively as possible, and multiple sensors are integrated [5]. We adjust and optimize the combined use of resources to maximize the use of the advantages and complementary functions of multisensor coordinated detection.

In the construction of large-scale construction projects, a single sensor cannot complete reliable measurements of the structure. With the development of sensor technology and information processing technology, multiple or multiple sensors can be used at the same time to perform multidimensional measurement of the same measurement object. Based on the deepening of CAD technology and the application of technological changes, BIM technology is the inevitable result of the development of information technology in the construction field to a higher stage and is considered by the international engineering community as an innovative technology for building productivity [6]. The application level of BIM in China is still shallow, mainly used for collision inspection, high-definition inspection, comprehensive pipeline deepening, and engineering quantity calculation, but the system has not yet been established [7]. The application of BIM in the construction schedule management of large-scale construction projects, in most cases, is limited to the virtual representation of the construction process, and the actual construction schedule management is rarely used [8]. According to project experience management, most construction companies mainly use traditional schedule management methods combined with actual websites. In short, although the application of BIM technology has many difficulties and obstacles, there are also many positive changes, creating a good background for the comprehensive application of BIM technology in the domestic construction industry [9]. For construction companies, the top priority is to apply BIM technology, including government policy regulations, owner requirements, fierce market competition, the needs of their own company operations, and social responsibility requirements [10].

## 2. Related Work

The literature proposes that multisensor information fusion originated from the necessity of the military C3I system, which was established in the 1980s. According to the literature, in the sensor network, if the information collected by different sensors is simply superimposed, the connection between the information of each sensor will be destroyed, and the amount of data is too much to be processed [11]. The literature suggests that BIM technology builds a virtual three-dimensional model on a computer, and through the use of digital technology, it provides a completely consistent database and information basis for the model [12]. The survey shows that the average timetable delay rate for construction projects is 55%. Multisensor coordinated detection technology is to acquire, identify, and process target environmental information through multiple sensors through wireless communication. It is proposed in the literature to send the information to the staff. Multisensor detection is an important area of collaboration technology. According to the literature, Bechtel has developed a graphical simulation tool 4D-Planner [13]. As a result, users can import progress status files and 3D models, realize the correlation between models and schedules, and automatically generate simulation files. In this way, the project manager can efficiently plan and manage the project [14]. The literature proposes that CIFE realizes the correlation between the 3D model of the computer and the progress of the construction, so that the construction sequence can be visualized [15].

## 3. Construction Schedule Management for Large-Scale Construction Projects

### 3.1. Traditional Construction Schedule Management Methods

Limited resources-shortest optimization construction period is to adjust the timetable under resource constraints and find the shortest construction period optimization method. When the amount of necessary resources exceeds the supply in a certain period of time, in order to eliminate the contradiction of resource restrictions, adjust the plan or allocate resources reasonably, in order to minimize the impact on the construction period, and increase the impact on the construction period. During the construction period, when the *T* project needs the KTH resource of job I-j at a specific time *T*, it also needs the *H*(*T*) task of the KTH resource and can be defined as

(1) $$\begin{array}{c} R^{k}\left(t\right)=\overset{H\left(t\right)}{\underset{1}{\sum }}\;r_{i-j}^{\left(k\right)}\left(t\right)\left(t\in T\right). \end{array}$$

The optimization of fixed resources during the project includes the minimum dispersion method, the minimum range method, and the peak cutting method. The minimum dispersion method is more widely used, and the optimization effect becomes higher. Therefore, this article only analyzes the application of the minimum dispersion method when optimizing schedule planning. Resource demand dispersion refers to the average of the sum of squares of the difference between resource demand and average demand in each period. The calculation formula is as follows:

(2) $$\begin{array}{c} \sigma^{2}=\frac{1}{T}\overset{T}{\underset{t=1}{\sum }}\;{\left(R_{t}-R_{m}\right)}^{2}=\frac{1}{T}\overset{T}{\underset{i=1}{\sum }}\;{\left(R_{t}-\frac{1}{T}\overset{T}{\underset{t=1}{\sum }}\;R_{t}\right)}^{2}=\frac{1}{T}\left(\overset{T}{\underset{t=1}{\sum }}\;R_{t}^{2}-\overset{T}{\underset{t=1}{\sum }}\;2R_{t}R_{m}+TR_{m}^{2}\right)=\frac{1}{T}\overset{T}{\underset{t=1}{\sum }}\;R_{t}^{2}-R_{m}^{2}. \end{array}$$

Because the project time is fixed, only unimportant tasks can be adjusted. Within the allowable range of the total time difference, the specific optimization methods and steps are as follows.

If work *i* − *j* starts on day *k* and ends on day 1, the resource requirements for that day will be fixed. Under certain circumstances, *i* − *j* will move after 1 day, that is,

(3) $$\begin{array}{c} R_{k}^{\prime }=R_{k}-\gamma_{i-j},\\ R_{l+1}^{\prime }=R_{l+1}+\gamma_{ij}. \end{array}$$

Then the variance of the variance is

(4) $$\begin{array}{c} \Delta \sigma^{2}=\frac{1}{T}\left\{\left[{\left(R_{k}\right)}^{2}+{\left(R_{l+1}^{\prime }\right)}^{2}\right]-\left[\left(R_{k}^{2}|+|R_{l+1}^{2}\right)\right]\right\}\\ =\frac{2}{T}\gamma_{i-j}\left[\left(R_{l+1}+\gamma_{i-j}\right)-R_{k}\right]. \end{array}$$

If (*R*_*l*+1_+*γ*_*i*−*j*_) − *R*_*k*_ ≥ 0, the postponement of work *I* − *J* does not affect the balanced allocation of resources, indicating that it is not appropriate to move one day later. In this case, you need to study whether you can move after 2 days. In case,

(5) $$\begin{array}{c} \left[\left(R_{l+1}+\gamma_{i-j}\right)-R_{k}\right]+\left[\left(R_{l+2}+\gamma_{i-j}\right)-R_{k+1}\right]<0. \end{array}$$

The important parameter used in optimization is the direct cost ratio. This is the ratio of the increase in direct cost per unit time of the work speeding up. Then the cost ratio is as follows:

(6) $$\begin{array}{c} e=\frac{C_{D}-C_{d}}{D-d}. \end{array}$$

**Figure 1:**
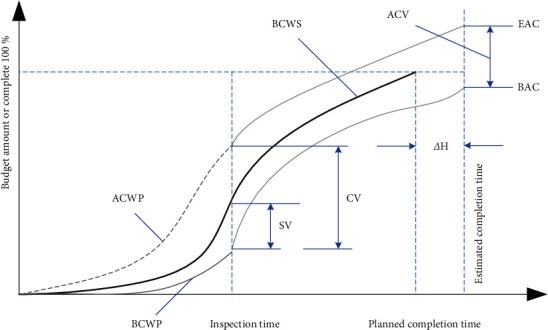
Evaluation curve of the earned value method.

The schedule deviation (SV) and schedule performance index (SPI) are used to analyze and control the schedule through the artistic value method. When SV is negative, progress is slow, and actual progress is behind the planned schedule. When SV is positive, it means that the progress is in progress, and the actual progress is faster than the planned progress. Similarly, in the case of SPI <1, progress is slow, and actual progress is slower than planned. SPI >1 means the progress is in progress, and the actual progress is faster than the planned progress. ΔH represents the estimated delay time when the project is completed.  Figure 1 shows the evaluation curve of the arch value method.

**Figure 2:**
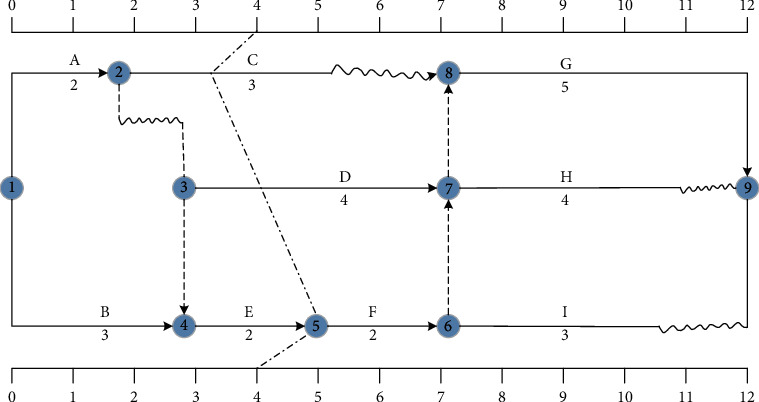
Comparison chart of forward line progress.

The forward line method refers to the method of comparing the actual progress of the project with the planned progress by drawing the forward line of the actual progress of the project within a certain inspection time and adopting corresponding measures to monitor and control the construction progress.  Figure 2 is a comparison chart of the progress of the forward line approach.

### 3.2. BIM Construction Management Optimization Model

**Figure 3:**
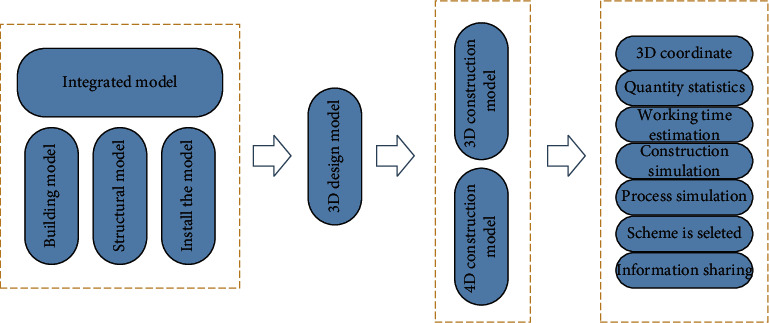
BIM-based schedule management implementation approach.

According to the characteristics of the project and the application program that can provide BIM software, the implementation method and framework of BIM in the process of the project are defined.  Figure 3 shows the implementation method of BIM-based schedule management.

In the process of project construction, there are many factors that affect the work efficiency, management level, drawing issues, construction quality, and other construction schedules of workers. By introducing BIM technology and using BIM's visualization, parameterization and other features, the impact of unfavorable factors on the construction schedule has been reduced. Therefore, before the project is implemented, the implementation framework of BIM construction schedule management must be planned, and the application of BIM in schedule management must be clarified.

The implementation of BIM is usually based on goals. First of all, you must define the project's BIM realization goal and select the corresponding BIM application according to the implementation goal. Since the goals of BIM directly affect the initial planning and preparation and need to be consistent with the actual project and the needs of the enterprise, the decision of the goal must be specifically measured. During the project implementation phase (construction plan simulation, collision check, project quantity calculation, and material management), Luban company summarized 106 BIM application points of the construction party. The core interconnection between enterprises is different, and the requirements for BIM and the goals that should be achieved are also different. Therefore, only by setting a reasonable BIM to achieve the goal and selecting the appropriate BIM application can the project succeed. The realization goal of BIM can be divided into three levels, including enterprise management, project management, and technology application. From the perspective of project management, Table 1 shows the purpose of BIM schedule management and the corresponding BIM application points.

The BIM model is a parametric 3D entity model, which parameterizes the entities and functions of all components and saves them in the database in digital form. The model object is the consolidated database. The BIM model includes the physical properties of the object and the size of the component, the positional relationship of related components in the model, the number of components in the model, and a series of component materials and decorative materials such as components. The 3D construction model is the basis for all management and construction applications that use BIM technology. Therefore, before using BIM in progress management, a BIM3D construction model with LOD300-LOD400 level details is required. Generally referred to as the BIM model, it refers to the BIM design model used in the design phase, bidding phase, project cost calculation, and other project phases of designing the display, which cannot meet the needs of the construction phase. In the case of a construction unit, there are three ways to obtain a design model. One is from the design unit.

Establishing the relationship between the plan and the BIM model is an important task when using BIM to manage the schedule. How to establish the connection quickly and conveniently between the plan and the BIM model is the most basic and urgent requirement of all construction personnel. Currently, there are a variety of software tools that can be used to build 4D construction models. This section selects Navisworks software and Luban SP (SchedulePlan) software, which are widely used in China, to analyze the construction method of 4D construction model.

First, the 3D model is imported into the Navisworks software, and then the schedule compiled with the Microsoft Project software is linked to the 3D model. In order to associate each component of the 3D model with each task item of the timetable, it is first necessary to collect the scattered components in the model to form an integrated component set. The name of the component collection as a work package corresponds to the name of the task item in the schedule. To associate the 3D model with the timetable, there are two methods. One is Microsoft Project, and the other is directly carried out by Navisworks.

Luban SP software has two different methods to build 4D build mode. One is to link the 3D model with the schedule after directly compiling the schedule with the software. The other is to import the schedule compiled by Microsoft or Excel software and associate it with the 3D model.

### 3.3. Multisensor Network Technology for Construction Projects

**Figure 4:**
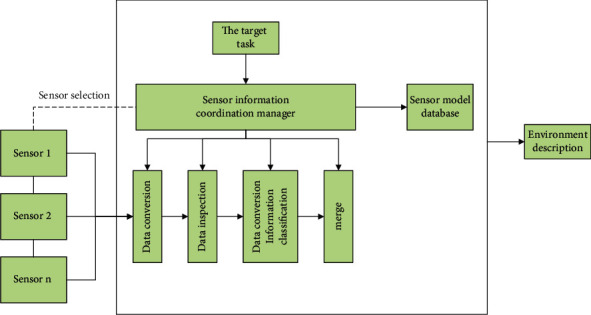
Multisensor information fusion process.

Multisensor information fusion refers to the use of computer technology to analyze and process the information collected by multiple sensors under a specific benchmark. This provides the basis for task decision-making and belongs to the integrated process of information processing. In recent years, the rapid and violent development of multisensor information fusion technology has boiled over and is widely used in criminal investigation, identification, surveillance, military, and other fields, as shown in Figure 4.

**Figure 5:**
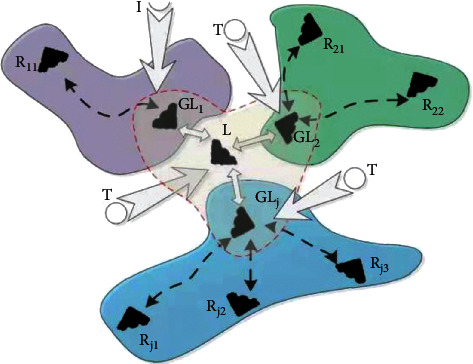
Schematic diagram of multisensor multitarget pairing.

The problem of target tracking was first raised in the military field used in battle on the battlefield. The principle is that the sensor is first used to search for the target area, after the target is found, the task is analyzed based on the target's location information and sensor resource information, and finally the algorithm and principle of matching between the sensor and the target are realized, and the target tracking task is completed.  Figure 5 is a schematic diagram of multisensor and multitarget pairing.

The target area environment is defined as a rectangle, and the grid lines are divided into *m* rows and *n* columns. According to the corresponding matrix area detection requirements, the corresponding area detection matrix refers to the expansion matrix of all sensors. Define the corresponding restriction matrix suitable for the deployment position of the sensor (river, lake, etc.). Next, the mathematical model of coordinated detection developed by multiple sensors is as follows:

(7) $$\begin{array}{c} \max f_{\left(x\right)}=\frac{\overset{m}{\underset{i=1}{\sum }}\;\overset{n}{\underset{j=1}{\sum }}\;y_{ij}}{m\times n}\text{,} \end{array}$$

where *y*_*ij*_ represents the elements of row *I* and column *j* of the area detection matrix.

Equality constraints:

(8) $$\begin{array}{c} Sum\;\left(X_{n\propto n}-Y_{m\times n}\right)=0\text{.} \end{array}$$

If *A* = *Y* − *E*, and the sum function represents the sum operation of all elements in the *A* matrix, then the element *a*_*ij*_ of the *A* matrix is defined as follows:

(9) $$\begin{array}{c} a_{ij}=\left\{\begin{array}{c} 1,\;ify_{ij}=1andx_{ij}=0,\\ 0,\;other \end{array}\right.\\ Sum\left(S_{m\times n}-T_{m\times n}\right)=0\text{.} \end{array}$$

If *B* = *s* − *t*, the aum function represents the sum operation of all elements in the *B* matrix, and the element *b*_*ij*_ of the *B* matrix is defined as follows:

(10) $$\begin{array}{c} b_{ij}=\left\{\begin{array}{c} 1if\;t_{ij}=0and\;s_{ij}=1,\\ 0other\text{.} \end{array}\right. \end{array}$$

After finding each extremum and global extremum, an individual in the particle swarm can use expression equation (11) to update its position and velocity, and determine the next search direction and step size.

(11) $$\begin{array}{c} x_{id}=x_{i}\frac{1}{d}v\text{.} \end{array}$$

When the environment of the main target area has no detection capability, and the expanded sensor node is not suitable for detection in this area, the fitness value of the particle becomes low. The formula of the evaluation function is as follows:

(12) $$\begin{array}{c} \max f_{\left(x\right)}=\frac{\overset{m}{\underset{i=1}{\sum }}\;\overset{n}{\underset{j=1}{\sum }}\;y_{ij}}{m\times n}-B_{1}Sum\left(X_{m\times n}-Y_{m\times n}\right)-B_{2}Sum\left(S_{m\times n}-T_{m\times n}\right)\text{.} \end{array}$$

The angle measured by the Volt-Angle Sensor No. *I* that is loaded at a certain moment is θ. Therefore, the deflection increment of the measuring point No. *I* is as follows:

(13) $$\begin{array}{c} \Delta d_{i\left(i-1\right)}=s_{i}\tan\;\theta_{i}1\leq i\leq N\text{.} \end{array}$$

In formula (13), when the deflection increment of the *i*-th measuring point is also used to express the deflection increments of other measuring points, the deflection of the *i*-th measuring point is similar to the Fibonacci sequence, and the following equation can be obtained:

(14) $$\begin{array}{c} d_{i\left(i-1\right)}=\overset{i}{\underset{j=1}{\sum }}\;\Delta d_{j\left(j-1\right)}=\overset{i}{\underset{j=1}{\sum }}\;s_{j}\tan\;\theta_{j}1\leq j\leq i\leq N\text{.} \end{array}$$

If the interval [*a*, *b*] has *N* + 1 points, Eon has a second continuous derivative in the interval [*a*, *b*]. Then:

(15) $$\begin{array}{c} S_{i}\left(x\right)=a_{i}x^{3}+b_{i}x^{2}+c_{i}x+d_{i}\left(i=1,2,\ldots n\right)\text{,}\\ S_{i}\left(x_{i}-0\right)=S_{i+1}\left(x_{i}+0\right)\left(i=1,2,\ldots n-1\right)\text{,}\\ S_{i}^{\prime }\left(x_{i}-0\right)=S_{i+1}^{\prime }\left(x_{i}+0\right)\left(i=1,2,\ldots n-1\right)\text{,}\\ S_{i}^{\prime \prime }\left(x_{i}-0\right)=S_{i+1}^{\prime \prime }\left(x_{i}+0\right)\left(i=1,2,\ldots n-1\right)\text{.} \end{array}$$

Each sensor only reflects a specific wavelength, so the external distortion value can be obtained based on the displacement of the Bragg wavelength returned by each sensor. The formula is as follows:

(16) $$\begin{array}{c} \lambda_{B}=2n_{eff}\wedge \text{.} \end{array}$$

With the change of the effective refractive index and period of light, the wavelength of the Bragg center will change. Then, the influence of temperature and strain on the Bragg wavelength can be expressed as follows:

(17) $$\begin{array}{c} \Delta \lambda_{B}=2n_{eff}\wedge \left[\left[1-\frac{n_{eff}}{2}\left(p_{12}-v\left(p_{11}+p_{12}\right)\right)\right]\varepsilon +\left[\alpha +\left(\frac{dn_{\text{eff}}/dT}{n_{\text{eff}}}\right)\Delta T\right]\right]\text{.} \end{array}$$

In the formula, *p*_11_, *p*_12_ are the elasticity coefficient and *v* is Poisson's ratio; then,

(18) $$\begin{array}{c} \Delta \lambda_{B}=2n_{eff}\wedge \left[\left(1-P_{e}\right)\varepsilon +\left(\alpha +\xi \right)\Delta T\right]\text{.} \end{array}$$

Equation (18) divides the same on both sides of the formula *λ*B has

(19) $$\begin{array}{c} \frac{\Delta \lambda_{B}}{\lambda_{B}}=\left(1-P_{e}\right)\varepsilon +\left(\alpha +\xi \right)\Delta T\text{.} \end{array}$$

Here, *P*_*e*_ is the effective elastic optical coefficient, *α* is the thermal expansion coefficient, and *ξ* is the thermal optical coefficient. Then, formula (19) is modified as follows:

(20) $$\begin{array}{c} \Delta \lambda_{B}=\lambda_{B}\left[\left(1-P_{e}\right)\varepsilon +\left(\alpha +\xi \right)\Delta T\right]=K_{e}\varepsilon +K_{T}\Delta T\text{.} \end{array}$$

Equation (20) shows that if the grating is only affected by temperature or distortion, the center wavelength deviation is linear, so the external temperature and distortion values can be calculated based on the wavelength deviation.

The principle of the effective independent method can be analyzed above, and the effective independent method is further analyzed from the perspective of formula derivation and applied in the research of this article. The idea of an effective independent method (EI) is as follows: according to the modal kinetic energy method (MKE), the candidate set of sensor positions is preselected. Starting from the minimum covariance benchmark of the modal coordinate estimation deviation, the degrees of freedom that contribute the least to the linear independence of the modal are sequentially deleted to obtain the most suitable sensor layout scheme for modal space estimation.

Define the sensor output response as *U*:

(21) $$\begin{array}{c} U_{s}=\Phi_{s}q+S\text{.} \end{array}$$

In formula (21), Φ is the modal matrix reduced according to the candidate measurement points, *n* is the degree of freedom, *N* is the modal order, *q* is the generalized modal coordinate, and *S* is the Gaussian white noise with dispersed *σ*2.

According to formula (21), the least square estimation of the most frequent coordinate *Q* can be obtained.

(22) $$\begin{array}{c} \bar{q}={\left[\Phi_{s}^{T}\Phi_{s}\right]}^{-1}\Phi_{s}^{T}U_{s}\text{.} \end{array}$$

According to equations (21) and (22), the covariance of *q* can be calculated as follows:

(23) $$\begin{array}{c} P=E\left[\left(q-\bar{q}\right){\left(q-\bar{q}\right)}^{T}\right]={\left(\frac{1}{\sigma^{2}}\Phi_{s}^{T}\Phi_{s}\right)}^{-1}\text{.} \end{array}$$

In formula (23), when *Q* reaches the maximum value, the covariance of *Q* is the smallest, and the characteristic formula of *Q* is as follows:

(24) $$\begin{array}{c} \left(Q-\lambda I\right)\Psi =0\text{.} \end{array}$$

According to matrix theory, matrix *E* can be composed as follows:

(25) $$\begin{array}{c} E=\Phi_{s}Q^{-1}\Phi_{s}^{T}=\Phi_{s}{\left(\Phi_{s}^{T}\Phi_{s}\right)}^{-1}\Phi_{s}^{T}\text{.} \end{array}$$

## 4. Design and Practical Application of the Construction Schedule Management System

### 4.1. System Data Collection Process Design

The LabVIEW data acquisition module is the lower end of the overall system architecture and the core of the health monitoring system. This module mainly completes the acquisition of sensor data, local storage and backup of data, and data display.

The drag-and-drop sensor data acquisition system describes the corresponding command according to the sensor address, reads the data in the buffer memory loop, converts the compressed BCD code, calculates the deflection, and displays the linear waveform, which can be roughly divided into such general steps. From data to waveform reading, according to the figure on the left of the red part, write the sensor read data command, change the address code sent to the 10 angle sensors, in hexadecimal format, cycle time 200 milliseconds, read data, and compare whether the checksum data are correctly judged. After the data stored in the array are collected (2000 milliseconds) and looped, the array data are looped, and then subsequent analysis and display are performed.

The grating sensor is the measurement of stress and strain. In order to calculate the final distortion value, it is necessary to solve the two problems of optical wavelength demodulation and wavelength conversion distortion value calculation. In this topic, use MOI's SM125 fiber grating demodulator to perform data transmission through the TCP port, and LabVIEW uses the TCP function of the data communication board to perform communication.

### 4.2. Database Design

The core function of the system is the display of sensor information, and it is necessary to design a table for initial storage of data. Choose SQLSERVER as a stable and reliable high-performance database management system, because it has a large-capacity data storage system, a variety of data, and long-term storage time period characteristics. Its characteristics are easy to use, high scalability, and highly integrated with related software. The design of the data table must be reasonable and comprehensive, so several data tables are as follows:

1. Table 2 shows the accelerometer data
2. Table 3 shows the sensor type table
3. Table 4 shows the data table of the tilt sensor

Database technology is used in many fields such as computer image processing, multimedia applications, and GIS. In order to run the application system stably and effectively, it is important to improve the performance of the database. In today's information age, high response speed and high-quality user expansion are particularly important. Database design optimization and adjustment, disk I/O optimization management, and query optimization are unique to certain aspects of database performance.

### 4.3. System Module Analysis

According to the actual needs of the project, through the integration of various technologies, the system will be divided into five modules for analysis and design: user management, sensor management, alarm management, data management, and system management. The subitems in the module are related to the content that the module needs to execute.

User management includes user information management and user rights management. User information management is the operation of users' personal information. User information management can be carried out on the information word, including the user's name, unit, region, user level, remarks, and other areas to be modified and deleted. User rights management is to classify user groups and control various levels through business permissions. The user's access rights are mainly divided into general users and administrator users.

Common user functions are as follows:

1. Display sensor information: this includes the display of sensor type groups, sensor specification attributes, real-time measurement, historical measurement, graphs, and trend analysis graphs.
2. Log view: various types of logs are generated during the operation, and users can view, download, or print them.
3. Alarm handling: Before the measurement, the alarm threshold is set for the sensor measurement of the program. When the measurement data exceed the threshold range, an alarm will be generated, and corresponding alarm information will be formed. Users can add, display, and delete alarm logs at the same time.

System user functions are as follows:

1. Display sensor information: can display all data and graphs.
2. Log view: can display the operation log records of general users and the background log of system operations. Can be downloaded or printed.
3. Alarm handling: this function is the same as for general users.
4. Change of parameter settings: in the design of the health monitoring system, the parameter setting interface is generally not described as fixed and unchangeable in the program. During the monitoring process, there are various reasons such as increasing the number of sensors and changing the measuring points of the sensors. System administrators can reconfigure parameters after physical changes, so the system can be updated in real time.

Sensor management includes the maintenance of sensor groups, the maintenance of sensor types, the maintenance of sensors, the change of upper and lower limits of sensors, and the replacement of sensors. For example, after the specification information of the sensor is input into the system, the detailed information of the sensor can be queried. Including number, type, upper and lower alarm value, measuring point location, manufacturing unit, specification, and model. The data management module includes real-time data display, time series, trend analysis, and other submodules. The data are displayed in the form of line graphs, bar graphs, pie graphs, and rose graphs. Taking real-time data display as an example, the submodule of real-time data display adopts high-definition lithography technology. Highcharts uses pure JavaScript descriptions with beautiful interfaces, does not need to execute plug-ins such as Flash and Java, has high compatibility, and supports most of the current browsers. There is a comprehensive API document containing 7 main functions in Highcharts.

### 4.4. System Simulation Analysis

**Figure 6:**
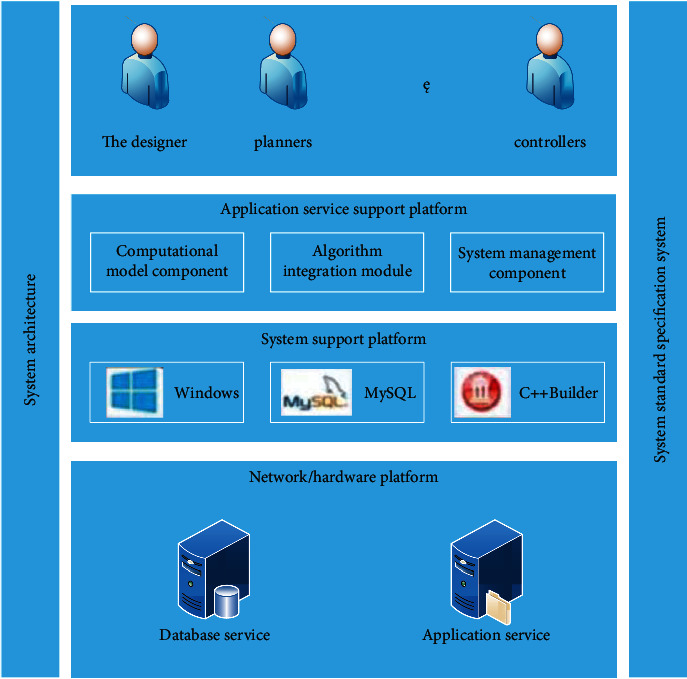
Schematic diagram of the prototype of the collaborative detection system.

This thesis is developed by C++Builder, and realizes the construction of sensor data management and coordinated detection platform through component development technology and database technology. The structured data between the systems are stored in the database, and the unstructured data (files) between the systems are stored in the file system. All important data of the system are formatted to use a multisensor coordinated detection system. The architecture of the coordinated detection system is shown in Figure 6.

The sensor coordination detection system is composed of multisensor target area environment recognition, multisensor deployment, multisensor information fusion, and multisensor target tracking. The design of a coordinated detection system needs to integrate many elements. For example, limited sensor resources and environmental factors in complex areas, and finally, until the adjustment of the sensor's action method and action parameters, the overall performance of the system is continuously optimized. The reasonable deployment of the sensor, the distribution of sensors and coordinates, and the coordinated detection system of matching are the most efficient the problem of multisensor and multiobjective to achieve full utilization of sensor resources under conditions. In this paper, combined with theoretical analysis and experimental simulation, the efficiency evaluation and optimization design of the multitarget sensor coordinated detection system are carried out.

System efficiency evaluation and system optimization design have four aspects: construction of efficiency evaluation index system, system efficiency evaluation modeling and analysis, system efficiency sensitivity analysis, and system structure optimization analysis. Among them, the system efficiency evaluation index system is constructed based on the analysis results of the system requirements and the system structure model. Select the evaluation index to define the cross-link relationship of the index. The modeling and analysis of system efficiency evaluation is based on the establishment of evaluation index system, system efficiency evaluation, and analysis efficiency evaluation model. Sensitivity analysis and structural optimization of system efficiency, based on the evaluation results, determine the main influencing factors, affect the degree of system efficiency, and provide the basis for architecture optimization. The platform of the coordinated detection system is developed by C++Builder, and the sensor data management and coordinated detection platform is realized by component development technology and database technology. The structured data between each system use the storage database, the unstructured data between the systems (documents) stored in the file system, and all the important data of the system after formatted into the multisensor coordinated detection system. The multisensor deployment operation is explained as follows, and the target tracking steps are similar. First, establish a key priority detection area. Then, set the number of sensors and the coverage radius. Finally, the deployment plan is optimized, the coordinates of the sensor's deployment node are saved in the database, and the final deployment plan of the sensor is displayed.

## 5. Conclusion

With the progress of society, economic development, and technological innovation, modern buildings have become larger and more complex, and the schedule management of engineering projects has become more complex, requiring a higher level of construction schedule management. Due to the limited technology, means, and methods of construction schedule management in the past, it is difficult to implement the schedule management schedule, which often results in the delay of the schedule. The emergence and development of BIM provides new ideas and technical means for construction schedule management. Through the application of BIM technology, problems such as the separation of plan and entity, the significant loss of project information, and the difficulty of tracking and controlling construction progress in traditional scheduling management can be solved, and the detailed management of construction progress can be realized and the level of construction management can be improved. The multisensor network coordinated detection technology based on the BIM model establishes a mathematical model by studying the multiplying effect of the multisensor detection technology as the research object, the multisensor deployment problem and the target tracking problem. Through related algorithms to verify the reliability and rationality of the model, finally a C++-based simulation system for sensor coordinated detection is proposed. Through continuous innovation in sensor technology and software technology, the development of a construction progress monitoring system is promoted. This paper proposes a construction schedule monitoring system based on multisensor information fusion, which analyzes and designs system hardware, software and fusion algorithms, and applies it to the construction schedule management system of large-scale construction projects.

## Figures and Tables

**Table 1:** Description of BIM schedule management implementation goals.

Target description	BIM application
Construction plan optimization	Construction simulation, construction section division
Site management	Construction site layout, temporary facility planning
Improve communication and coordination and information sharing	Information sharing platform established

**Table 2:** Acceleration sensor data sheet.

Field name	Description	Types of	Length
ID	Record ID (primary key)	Integer	—
POINT_ID	Measuring point ID	NVarChar	50
DIRECTION_X	Acceleration value in *X* direction	Double	—
DIRECTION_Y	*Y* direction acceleration value	Double	—
DIRECTION_Z	Acceleration value in *Z* direction	Double	—
DATE	Measure time	Date	—

**Table 3:** Sensor type table.

Field name	Description	Types of	Length
ID	Record ID (primary key)	Integer	—
NAME	Name	NVarChar	50
TABLENAME	Table name	NVarChar	50
SHOWTYPE	Data display type	Integer	—
TUTYPE	Icon type	Integer	—

**Table 4:** Data sheet of inclination sensor.

Field name	Description	Types of	Length
ID	Record ID (primary key)	Integer	—
POINT_ID	Measuring point ID	NVarChar	50
BENDING_VALUE	Deflection value of the first inclinometer	Double	—
DATE	Measure time	Date	—

## Data Availability

The data used to support the findings of this study are available from the corresponding author upon request.
